# Deconstructing sex differences in emotion regulation: the underlying role of gender attitudes

**DOI:** 10.3389/fpsyg.2026.1900243

**Published:** 2026-07-20

**Authors:** Daniel Oleas, Mirian Chicaiza-Briones, Tamara Cobo, Ciara M. Greene, Jose A. Rodas

**Affiliations:** 1 Department of Research, Universidad Ecotec, Samborondón, Ecuador; 2 Department of Social Anthropology, Basic Psychology and Public Health, Universidad Pablo de Olavide, Dos Hermanas, Sevilla, Spain; 3 School of Psychology, Universidad Espíritu Santo, Samborondón, Ecuador; 4 School of Psychology, University College Dublin, Belfield Campus, Dublin, Ireland

**Keywords:** cognitive reappraisal, Ecuadorian university students, emotion regulation, expressive suppression, gender role attitudes, gender differences, sex differences

## Abstract

**Introduction:**

Although biological sex is frequently associated with distinct emotion regulation profiles, this dichotomy often overlooks the influence of sociocultural constructs. This study aimed to determine whether specific gender role attitudes predict the use of cognitive reappraisal and expressive suppression, and whether biological sex moderates these relationships.

**Methods:**

A cross-sectional sample of 201 Ecuadorian university students completed the Gender Role Attitudes Scale and the Emotion Regulation Questionnaire. Data were analysed using multiple linear regressions and moderation analyses, controlling for income, mental health history, and marital status.

**Results:**

Biological sex neither significantly differentiated the use of cognitive reappraisal and expressive suppression, nor moderated the relationship between gender beliefs and these strategies. Following adjustments for multiple comparisons, non-egalitarian gender roles significantly and negatively predicted the use of both cognitive reappraisal and expressive suppression, whereas adherence to traditional roles positively predicted only the use of expressive suppression.

**Discussion:**

The findings suggest that the internalisation of sociocultural gender beliefs accounts for individual differences in specific emotion regulation strategies. Emotion regulation appears to be driven by perceived social permissibility, offering additional explanatory value beyond biological sex. Consequently, the promotion of egalitarian gender beliefs may be associated with increased psychological flexibility and adaptive emotional functioning.

## 1 Introduction

Emotion regulation refers to the heterogeneous set of automatic and controlled processes by which individuals influence the emotions they have, when they have them, and how they experience and express these feelings (Gross, 2015a). Within the process model of emotion regulation (Gross, 2015b), two of the most widely investigated strategies are cognitive reappraisal and expressive suppression. Cognitive reappraisal is an antecedent-focused strategy that involves construing a potentially emotion-eliciting situation in a way that changes its emotional impact. Conversely, expressive suppression is a response-focused strategy involving the deliberate inhibition of emotion-expressive behaviour once an emotional response has already been generated. An extensive body of literature has robustly demonstrated that the habitual use of cognitive reappraisal is associated with healthier patterns of affect, improved interpersonal functioning, and greater psychological well-being. In contrast, reliance on expressive suppression is frequently correlated with increased negative affect, heightened physiological arousal, and a greater vulnerability to psychopathology, including depression and anxiety disorders (Aldao et al., 2010; García-Fernández et al., 2025; Rodas et al., 2022). Consequently, identifying the individual differences that dictate the selection and implementation of these specific strategies remains critical for both clinical and developmental psychology.

Among the individual differences influencing emotional processing, biological sex and gender socialisation have garnered some empirical attention. Epidemiological data consistently reveal marked disparities in the prevalence of psychiatric conditions, with women exhibiting significantly higher rates of internalising disorders, such as depression and anxiety, and men displaying higher rates of externalising problems, including substance abuse and antisocial behaviours (Hartung and Lefler, 2019; Nolen-Hoeksema, 2012). Researchers have increasingly found that these disparities are partially mediated by differences in how men and women regulate their emotions. Empirical investigations indicate that women generally report using a wider repertoire of emotion regulation strategies compared to men (Goubet and Chrysikou, 2019). Furthermore, women are consistently found to engage more frequently in rumination, a maladaptive strategy that exacerbates depressive symptomatology (Alvarado-Zurita and Rodas, 2024; Nolen-Hoeksema and Aldao, 2011). Event-related potential (ERP) studies suggest that women may exhibit greater early emotional reactivity to negative stimuli, which subsequently influences their deployment of cognitive resources during regulation efforts (Gardener et al., 2013).

Conversely, the literature on men’s emotion regulation suggests a stronger tendency towards expressive suppression and the utilisation of externalising behaviours as a means of affective avoidance (Berke et al., 2018). Interestingly, neuroimaging studies suggest that while men and women may achieve comparable reductions in negative affect via cognitive reappraisal, men might execute this down-regulation more efficiently or automatically, evidencing lesser prefrontal cortex activation and greater amygdala attenuation (McRae et al., 2008). Despite these physiological and behavioural findings, biological sex alone provides an incomplete framework for understanding the nuances of emotion regulation, requiring a deeper evaluation of the sociocultural constructs that shape emotion regulation.

To evaluate the sociocultural constructs that shape emotion regulation, conceptual clarity is required regarding related terms. Biological sex refers to innate physiological characteristics. Gender identity denotes an individual’s internal sense of their gender. Gender socialisation represents the developmental process through which individuals learn the socially constructed behaviours and expectations a culture considers appropriate. This socialisation begins in early childhood and dictates the display rules regarding which emotions are acceptable to express and which must be concealed (Pejičić, 2024). Furthermore, gender role attitudes reflect the specific, internalised beliefs an individual holds regarding these societal norms, operating independently of, yet interacting with, biological sex. Traditional masculine norms frequently prescribe emotional stoicism, dominance, and the restriction of vulnerable emotions such as sadness or fear. This often leads men to default to expressive suppression or externalising coping mechanisms to avoid the social sanctions associated with violating these norms (Berke et al., 2019). Because masculinity is socially viewed as a status that must be continually earned and defended, men may experience chronic discrepancy stress when their emotional experiences conflict with rigid gender expectations, thereby reinforcing rigid internally mediated regulatory efforts like suppression (Berke et al., 2018). Conversely, traditional feminine norms often permit a wider range of emotional expression and encourage relational, emotion-focused coping. While fostering social support, this socialisation can also increase vulnerability to co-rumination and internalising distress (Nolen-Hoeksema, 2012). Difficulties in emotion regulation thus act as a critical transdiagnostic mechanism linking restrictive gender socialisation to subsequent psychopathology.

Consequently, an individual’s internalised gender role attitudes—their beliefs regarding the appropriateness of traditional versus egalitarian gender roles—are likely to serve as cognitive schemas that directly govern their emotion regulation efforts. Gender beliefs should operate as a proximal factor linking distal societal socialisation to an individual’s immediate cognitive and emotional responses. Individuals who strongly endorse traditional gender roles may feel compelled to regulate their emotions in strict accordance with stereotypical expectations, potentially over-relying on expressive suppression to mask counter-normative feelings. Conversely, individuals who hold egalitarian (non-traditional) gender beliefs may experience greater psychological flexibility, perceiving fewer social constraints on their emotional experiences. This cognitive flexibility, which has been shown to mitigate anxiety and depressive symptoms (García-Fernández et al., 2025), may facilitate the use of more adaptive, antecedent-focused strategies like cognitive reappraisal, allowing the individual to reframe emotional events without the rigid imperative to simply hide their reactions.

The interplay between gender beliefs and emotion regulation is particularly salient within Latin American contexts, where traditional cultural scripts such as machismo and marianismo have historically dictated rigid gender paradigms associated with higher levels of negative cognitions and emotions (Nuñez et al., 2016). Machismo emphasises male dominance, emotional restriction, and self-reliance, whereas marianismo idealises female submissiveness, self-sacrifice, and emotional nurturance. However, contemporary Latin American university populations are undergoing significant sociocultural transitions, increasingly challenging these traditional archetypes in favour of more egalitarian perspectives. Recent research within Ecuador has begun to map the contours of emotion regulation in these populations, noting that while traditional gender differences in strategies like rumination and self-blame persist under high-stress conditions (Alvarado-Zurita and Rodas, 2024), the overarching influence of shifting cognitive paradigms remains complex. Understanding how the endorsement of egalitarian versus traditional gender beliefs influences the selection of specific emotion regulation strategies within this transitional cultural background is critical for elucidating the underlying sources of variability in emotional processing between men and women.

### 1.1 The present study

While previous research has established a broad connection between general gender beliefs and emotion regulation, the specific mechanics of this relationship remain underexplored. Specifically, it is unclear which dimensions of gender role attitudes drive these regulatory strategies and how these dynamics might differ between men and women when accounting for complex life contexts. Therefore, the present study aims to deepen this understanding through three primary objectives: (1) to identify which specific domains of gender role attitudes (e.g., egalitarian, traditional, or marital roles) most strongly predict the use of cognitive reappraisal and expressive suppression; (2) to determine if sex moderates the relationship between these gender beliefs and emotion regulation strategies; and (3) to evaluate whether these associations persist when controlling for relevant demographic covariates, such as mental health history, relationship status, and socioeconomic factors.

## 2 Methodology

### 2.1 Participants

The sample consisted of 201 university students from Ecuador, recruited using non-probability convenience and snowball sampling methods. The sample comprised 127 women (63.2%) and 74 men (36.8%). Participants were aged between 18 and 25 years (M = 20.9, SD = 2.64). Regarding marital status, most participants were single (86.6%, *n* = 174), followed by married participants (7.5%, *n* = 15) and those cohabiting with a partner (5.5%, *n* = 11). In terms of academic background, students were enrolled in various degree programmes, most commonly classified as other fields of study (62.7%, *n* = 126), followed by Psychology (28.9%, *n* = 58), Architecture (5.5%, *n* = 11), and Medicine (2.5%, *n* = 5). Monthly household income was distributed as follows: USD 266–639 (37.3%, *n* = 75), USD 640–1,423 (20.9%, *n* = 42), USD 1,424–3,909 (16.9%, *n* = 34), USD 3,910 or more (12.4%, *n* = 25), and less than USD 100 (12.4%, *n* = 25). All participants provided informed consent prior to data collection. At the time of data collection, the basic family basket in Ecuador was valued at USD 798.31 per month (Instituto Nacional de Estadística y Censos, 2025). Based on self-reported clinical history, most participants reported no previous psychiatric diagnosis (91.0%, *n* = 183). Inclusion criteria were being aged 18 years or older, being enrolled as a university student in Ecuador, understanding Spanish, and providing informed consent prior to participation. Participants were excluded if they did not provide informed consent or reported being under the influence of psychoactive substances at the time of participation.

### 2.2 Instruments

#### 2.2.1 Gender Role Attitudes Scale (GRAS)

The Gender Role Attitudes Scale (GRAS), developed by Zeyneloğlu and Terzioğlu (2011), was used to evaluate participants’ beliefs, values, and social expectations regarding the roles men and women should adopt across various life domains (e.g., family, work, and social environments). This scale consists of 38 items distributed across five dimensions: egalitarian gender roles (e.g., Spouses should decide together in the family), women’s roles (e.g., A woman’s basic task is motherhood), marriage roles (e.g., Husband’s cheating on a wife should be regarded as normal), traditional roles (e.g., A man’s main task in the house is breadwinning), and men’s roles (e.g., Education level of the man should be higher than woman in marriages). All sub-scales include 8 items, except for male roles which only includes 6. Responses are recorded on a 5-point Likert scale ranging from 1 (strongly disagree) to 5 (strongly agree). Since in most scales higher scores represent more traditional (less egalitarian) attitudes, the scoring from the first scale, items 3, 4 and 6 from the 2nd scale and item 4 from the 3rd scale were reversed. In this way, the first scale has been relabelled as ‘non-egalitarian gender roles’. Individual scores are calculated for each dimension, by summing the response given to each item, as well as a scale’s total scores ranging from 38 to 190, with higher scores indicating less egalitarian attitudes toward gender roles in all cases. The instrument has demonstrated strong psychometric properties, with original validation studies reporting a high reliability coefficient of 0.92. In the present sample, the subscales demonstrated acceptable to excellent internal consistency, with Cronbach’s alphas ranging from 0.67 to 0.89 and McDonald’s *ω* ranging from 0.68 to 0.90 (non-egalitarian roles: *α* = 0.89, *ω* = 0.90; women’s roles: *α* = 0.67, *ω* = 0.68; marriage roles: *α* = 0.84, *ω* = 0.84; traditional roles: *α* = 0.81, *ω* = 0.81; men’s roles: *α* = 0.81, *ω* = 0.81).

#### 2.2.2 Emotion regulation questionnaire (ERQ)

To measure emotion regulation strategies, the study employed the Emotion Regulation Questionnaire (ERQ), originally developed by Gross and John (2003). Specifically, the Spanish adaptation validated in Ecuador by Moreta-Herrera et al. (2022) was used. This self-report measure evaluates two primary emotion regulation strategies through 10 items: cognitive reappraisal (6 items) and expressive suppression (4 items). Participants rate the frequency with which they use these strategies in their daily lives using a 7-point Likert scale ranging from 1 (strongly disagree) to 7 (strongly agree). An average score was calculated for each subscale, where higher scores indicate a more prevalent use of the strategy. The Ecuadorian validation of the ERQ has shown excellent internal consistency, with reliability coefficients reaching 95% for both the cognitive reappraisal and expressive suppression subscales. In the current study, both subscales displayed strong internal consistency, yielding a Cronbach’s *α* of 0.90 and a McDonald’s *ω* of 0.90 for cognitive reappraisal, and an *α* of 0.83 and an *ω* of 0.83 for expressive suppression.

### 2.3 Procedure

The study was promoted to students through both virtual and in-person announcements within their classrooms. Prior to completing the survey, all participants were informed about the study’s primary objectives. The research strictly adhered to the ethical guidelines established by the American Psychological Association (2017), ensuring voluntary participation, informed consent, and data confidentiality. To further protect participant privacy and anonymity, unique codes were assigned to each individual’s data entry. Ethical approval was granted by Ecotec research board (No 26-11-11-2025).

Data collection took place between December 22, 2025, and May 7, 2026. Participants completed the demographic questionnaire, the GRAS, and the ERQ online via the Google Forms platform. To encourage participation, students were offered course credit. The online administration of the instruments took approximately 15–20 min per participant to complete.

### 2.4 Analyses

All statistical analyses were conducted using R software (R Core Team, 2024), with the *car* (Fox and Weisberg, 2019), *dplyr* (Wickham et al., 2026), and *psych* (Revelle, 2026) packages. Preliminary analyses included descriptive statistics to characterise the sample across demographic and primary psychological variables. Items’ mean scores were used in all calculations. To evaluate differences in emotion regulation strategies and gender role attitudes based on biological sex, independent samples Welch’s *t*-tests were performed, with Cohen’s *d* calculated to determine effect sizes. To address the primary research aims, a series of linear regression models were built. First, multiple linear regression analyses were conducted to identify which specific dimensions of the GRAS significantly predicted cognitive reappraisal and expressive suppression. Prior to interpreting these models, the assumption of multicollinearity was assessed using the Variance Inflation Factor (VIF). Subsequently, to examine whether biological sex moderated the relationship between gender beliefs and emotion regulation, moderation analyses were performed by introducing interaction terms (Sex × GRAS dimensions) into the regression models. Finally, three-step hierarchical multiple regression analyses were conducted to determine if the predictive utility of the significant GRAS dimensions persisted when controlling for relevant demographic covariates (income, mental health history, and marital status). Covariates were entered in Block 1, biological sex in Block 2, and the significant GRAS dimensions in Block 3, with model improvement evaluated via the change in *F*-statistic (*ΔF*) and explained variance (*R*^2^). To account for the increased risk of Type I error associated with conducting multiple statistical tests, a Holm–Bonferroni correction was applied to the *p*-values of the primary regression models.

## 3 Results

### 3.1 Descriptive statistics and comparisons by sex

Descriptive statistics were calculated to examine the distributions of the primary psychological variables (see Table 1). Overall, participants reported a moderate use of emotion regulation strategies, with cognitive reappraisal being endorsed more frequently than expressive suppression. Regarding gender beliefs the sample demonstrated variability across all five dimensions of the GRAS.

**Table 1:** Descriptive statistics and sex differences for primary psychological variables.

Variable	Female M (SD)	Male M (SD)	*t*	*p*	Cohen’s *d*
Emotion regulation					
Cognitive reappraisal	4.98 (1.36)	4.75 (1.31)	1.17	0.242	0.17
Expressive suppression	4.33 (1.53)	4.33 (1.28)	−0.00	0.996	0.00
Gender beliefs					
GRAS 1: non-egalitarian roles	1.57 (0.78)	1.69 (0.46)	−1.39	0.165	0.18
GRAS 2: women’s roles	2.47 (0.67)	2.76 (0.56)	−3.25	0.001	0.46
GRAS 3: marriage roles	1.87 (0.86)	2.53 (0.89)	−5.09	< 0.001	0.76
GRAS 4: traditional roles	2.62 (0.88)	3.11 (0.78)	−4.17	< 0.001	0.59
GRAS 5: men’s roles	2.11 (0.90)	2.55 (0.88)	−3.45	0.001	0.50

Higher scores on the GRAS dimensions indicate a less egalitarian (more traditional) role endorsement.

Independent samples Welch’s *t*-tests were performed to examine potential differences between women and men across emotion regulation strategies and gender beliefs (results in Table 1). Regarding emotion regulation, no significant differences were observed between sexes, with participants reporting statistically similar levels of cognitive reappraisal and expressive suppression. In contrast, significant sex differences emerged across four of the five dimensions of the GRAS, with no significant difference observed for non-egalitarian roles. Men scored significantly higher than women on women’s roles, marriage roles, traditional roles, and men’s roles. These findings indicate that biological sex robustly differentiates specific ideological gender beliefs within this group of Ecuadorian university students, even though it does not differentiate their emotion regulation habits.

Additionally, intercorrelations among the GRAS dimensions were examined (see Table 2). The results showed positive associations among all subscales, ranging from small-to-moderate to strong in magnitude (*r* = 0.26–0.70), with all correlations reaching statistical significance (*p* < 0.001).

**Table 2:** Zero-order correlations among GRAS dimensions.

Variable	1	2	3	4	5
1. GRAS 1 (non-egalitarian roles)	—				
2. GRAS 2 (women’s roles)	0.44	—			
3. GRAS 3 (marriage roles)	0.51	0.63	—		
4. GRAS 4 (traditional roles)	0.26	0.56	0.55	—	
5. GRAS 5 (men’s roles)	0.45	0.59	0.70	0.65	—

### 3.2 Multiple regression analyses of gender belief dimensions

To identify which specific domains of gender role attitudes most strongly predict emotion regulation strategies, two multiple linear regression analyses were conducted (see Table 3). Prior to interpreting the regression models, diagnostic analyses were conducted to evaluate core statistical assumptions. The assumption of multicollinearity was satisfied, with Variance Inflation Factor (VIF) values ranging from 1.45 to 2.54. Homoscedasticity was confirmed across all models using the Breusch–Pagan test (all *p* > 0.05). The Shapiro–Wilk test indicated that residuals for the expressive suppression models were normally distributed (all *p* > 0.05). Although residuals for the cognitive reappraisal models significantly deviated from strict normality (Aim 1: *p* < 0.001; Block 3: *p* = 0.006), linear regression estimators remain robust to such violations given the sample size. Finally, Cook’s distance was computed to screen for influential observations. Whilst approximately 7% of cases exceeded the conservative 4/N threshold across the models, the maximum Cook’s distance observed was 0.21, which falls well below the standard cut-off of 1.0. Consequently, no single observation was deemed to exert undue influence on the model parameters.

**Table 3:** Multiple linear regressions predicting emotion regulation strategies from GRAS dimensions.

Variable	*B*	SE	*β*	*t*	*p*
Model 1: cognitive reappraisal					
Constant	35.41	2.32		15.27	<0.001
GRAS 1 (non-egalitarian roles)	−4.30	0.92	−0.36	−4.69	<0.001
GRAS 2 (women’s roles)	−0.98	1.13	−0.08	−0.87	1
GRAS 3 (marriage roles)	−0.71	0.88	−0.08	−0.80	1
GRAS 4 (traditional roles)	1.94	0.83	0.21	2.34	0.143
GRAS 5 (men’s roles)	−0.23	0.90	−0.03	−0.25	1
Model 2: expressive suppression					
Constant	15.58	1.69		9.19	<0.001
GRAS 1 (non-egalitarian roles)	−2.66	0.67	−0.32	−3.97	<0.001
GRAS 2 (Women’s Roles)	0.47	0.83	0.05	0.56	1
GRAS 3 (marriage roles)	−0.45	0.64	−0.07	−0.70	1
GRAS 4 (traditional roles)	1.96	0.61	0.30	3.23	0.012
GRAS 5 (men’s roles)	0.12	0.66	0.02	0.19	1

*B* = unstandardised regression coefficient; SE = standard error; *β* = standardised regression coefficient. Higher scores on the GRAS dimensions indicate a less egalitarian (more traditional) role. Model 1: *R*^2^ = 0.18, adjusted *R*^2^ = 0.16. Model 2: *R*^2^ = 0.15, adjusted *R*^2^ = 0.12.

The overall first model was statistically significant, *F*(5, 195) = 8.78, *p* < 0.001, explaining 18.4% of the variance (Adjusted *R*^2^ = 0.16) in cognitive reappraisal. An examination of the individual coefficients revealed that non-egalitarian gender roles (GRAS 1) significantly and negatively predicted cognitive reappraisal, indicating that less egalitarian attitudes were associated with a lower use of reappraisal. Conversely, attitudes toward traditional roles (GRAS 4) emerged as a significant positive predictor. Beliefs concerning women’s roles, marriage roles, and men’s roles did not significantly predict cognitive reappraisal. For the second model evaluating expressive suppression, the overall equation was also significant, *F*(5, 195) = 6.69, *p* < 0.001, accounting for 14.6% of the variance (Adjusted *R*^2^ = 0.12). The pattern of significant predictors mirrored the first model. Non-egalitarian roles (GRAS 1) significantly and negatively predicted expressive suppression, whilst traditional roles (GRAS 4) positively predicted it.

### 3.3 Moderation analysis: the role of biological sex

To determine whether sex moderated the relationship between gender role beliefs and emotion regulation strategies, a series of moderation analyses were conducted using multiple linear regression. Interaction terms were created between sex, coded as female = 0 and male = 1, and the gender belief predictors.

Initial models examined whether sex moderated the association between the total GRAS score and each emotion regulation strategy. For cognitive reappraisal, the overall model was statistically significant, *F*(3, 197) = 4.18, *p* = 0.007, explaining 6.0% of the variance (Adjusted *R*^2^ = 0.046). The main effect of the total GRAS score was significant (*B* = −0.12, *p* = 0.001), whereas the main effect of sex (*B* = −1.07, *p* = 0.185) and the interaction between total GRAS score and sex (*B* = 0.09, *p* = 0.189) were not significant. For expressive suppression, the overall model was not statistically significant, *F*(3, 197) = 0.51, *p* = 0.679, explaining 0.8% of the variance (Adjusted *R*^2^ = −0.007). Neither the total GRAS score (*B* = −0.02, *p* = 0.708), sex (*B* = −1.03, *p* = 0.244), nor their interaction (*B* = 0.08, *p* = 0.239) reached statistical significance.

Given that the previous regression analyses identified non-egalitarian roles (GRAS 1) and traditional roles (GRAS 4) as the main predictors of emotion regulation, follow-up moderation models were conducted using these two dimensions as focal predictors. Results from these models are presented in Table 4.

**Table 4:** Moderation analyses of sex on the relationship between significant gras dimensions and emotion regulation.

Variable	*B*	SE	*t*	*p*
Model 2c: cognitive reappraisal				
Constant	5.89	0.38	15.71	<0.001
GRAS 1 (non-egalitarian roles)	−0.90	0.14	−6.27	<0.001
Sex (male)	−1.06	0.79	−1.35	0.179
GRAS 4 (traditional roles)	0.19	0.13	1.48	0.141
GRAS 1 × sex	0.37	0.36	1.04	0.300
GRAS 4 × sex	0.07	0.23	0.31	0.761
*R*^2^ = 0.18, adjusted *R*^2^ = 0.16, *F*(5, 195) = 8.84, *p* < 0.001				
Model 2d: expressive suppression				
Constant	4.16	0.41	10.13	<0.001
GRAS 1 (non-egalitarian roles)	−0.72	0.16	−4.56	<0.001
Sex (male)	−0.82	0.86	−0.95	0.344
GRAS 4 (traditional roles)	0.49	0.14	3.52	<0.001
GRAS 1 × sex	0.26	0.39	0.67	0.502
GRAS 4 × sex	0.07	0.26	0.27	0.788
*R*^2^ = 0.15, adjusted *R*^2^ = 0.13, *F*(5, 195) = 6.87, *p* < 0.001				

*B* represents unstandardised regression coefficients. GRAS, Gender Role Attitudes Scale. Female is the reference category for sex.

For cognitive reappraisal, the targeted model including GRAS 1, GRAS 4, sex, and their interaction terms was statistically significant. However, this effect was driven by the main effect of non-egalitarian roles (GRAS 1). The main effect of traditional roles (GRAS 4), the main effect of sex, and the interaction terms between sex and both GRAS dimensions were not statistically significant. Thus, sex did not moderate the association between these gender role beliefs and cognitive reappraisal.

For expressive suppression, the targeted model was also statistically significant. In this model, both non-egalitarian roles (GRAS 1) and traditional roles (GRAS 4) retained significant main effects. However, the main effect of sex and the interaction terms between sex and both GRAS dimensions were not statistically significant. Therefore, sex did not moderate the association between gender role beliefs and expressive suppression. Overall, the moderation analyses provided no evidence that the relationship between gender role beliefs and emotion regulation differed between women and men.

### 3.4 Hierarchical regression: controlling for demographic covariates

We also evaluated whether the associations between specific gender beliefs and emotion regulation strategies persist when controlling for relevant clinical and demographic variables. For this, two three-step hierarchical multiple regression analyses were conducted predicting cognitive reappraisal and expressive suppression. Based on the findings from the initial dimension-level analyses, significant GRAS dimensions were isolated as the primary predictors. Demographic covariates (income, mental health history, and marital status) were entered in Block 1, biological sex was added in Block 2, and the targeted GRAS dimensions were entered in the final step (Block 3). One participant was excluded from these analyses due to missing data regarding marital status.

For both cognitive reappraisal and expressive suppression, the addition of demographic covariates in Block 1 and biological sex in Block 2 did not result in statistically significant improvements to the models. However, the inclusion of the targeted gender belief dimensions in the final block (Block 3) produced highly significant overall models for both emotion regulation strategies. The final model for cognitive reappraisal accounted for 26.8% of the total variance, with the GRAS dimensions significantly improving predictive power over the baseline covariates and sex, *ΔF*(2, 189) = 20.92, *p* < 0.001. Similarly, the final model for expressive suppression accounted for 18.1% of the total variance, demonstrating a significant improvement in predictive power in the final step, *ΔF*(2, 189) = 17.17, *p* < 0.001. Results from both final models are presented in Table 5.

**Table 5:** Hierarchical regressions predicting emotion regulation strategies controlling for covariates (final models).

Variable	Cognitive reappraisal			Expressive suppression		
	*B*	*β*	*p*	*B*	*β*	*p*
Constant	5.97 [5.17, 6.77]	—	<0.001	3.93 [3.02, 4.83]	—	<0.001
Income ($1,424–$3,909)	−0.04 [−0.67, 0.59]	−0.01	0.900	0.17 [−0.54, 0.88]	0.04	0.637
Income ($266–$639)	−0.52 [−1.10, 0.05]	−0.19	0.070	0.10 [−0.55, 0.75]	0.03	0.763
Income ($640–$1,423)	−0.24 [−0.85, 0.37]	−0.07	0.440	0.33 [−0.36, 1.03]	0.09	0.345
Income (<$100)	−0.84 [−1.51, −0.16]	−0.21	0.020	0.25 [−0.52, 1.01]	0.06	0.527
Mental health (yes)	0.06 [−0.56, 0.68]	0.01	0.850	−0.04 [−0.74, 0.66]	−0.01	0.902
Marital status (other)	−2.98 [−5.41, −0.54]	−0.16	0.020	−1.67 [−4.44, 1.09]	−0.08	0.234
Marital status (free union)	−0.92 [−1.68, −0.17]	−0.16	0.020	−0.89 [−1.75, −0.04]	−0.14	0.041
Marital status (married)	0.00 [−0.68, 0.68]	0.00	>0.999	0.05 [−0.72, 0.82]	0.01	0.901
Sex (male)	−0.29 [−0.66, 0.08]	−0.10	0.120	−0.24 [−0.65, 0.18]	−0.08	0.268
GRAS 1 (non-egalitarian roles)	−0.84 [−0.58, −0.31]	−0.43	<0.001	−0.72 [−0.53, −0.22]	−0.34	<0.001
GRAS 4 (traditional roles)	0.29 [0.04, 0.26]	0.19	0.010	0.55 [0.16, 0.42]	0.33	<0.001

*B* represents unstandardised regression coefficients and *β* the standardised coefficient. CI, Confidence Interval. Cognitive reappraisal model fit: *R*^2^ = 0.270, Adjusted *R*^2^ = 0.223, *F*(12, 188) = 5.79, *p* < 0.001. Expressive suppression model fit: *R*^2^ = 0.181, Adjusted *R*^2^ = 0.134, *F*(11, 189) = 3.81, *p* < 0.001. The reference category for Income is “≥$3,910.” The reference category for mental health is “no.” The reference category for marital status is “single.” The reference category for sex is “female.” Higher scores on the GRAS dimensions indicate a less egalitarian (more traditional) role.

In the final models predicting cognitive reappraisal and expressive suppression, non-egalitarian roles (GRAS 1) and traditional roles (GRAS 4) emerged as significant robust predictors for both. Non-egalitarian roles was a significant negative predictor, indicating that lower egalitarian attitudes were associated with reduced use of reappraisal and expressive suppression. Conversely, traditional roles acted as a significant positive predictor for both emotion regulation strategies. Furthermore, specific demographic factors showed significant effects on cognitive reappraisal: participants earning less than $100 and those in a free union reported significantly lower cognitive reappraisal compared to their respective reference groups. For expressive suppression, being in a free union was the only demographic covariate to demonstrate a significant negative association.

## 4 Discussion

The primary objective of the present study was to deepen the understanding of the relationship between specific gender role attitudes and emotion regulation strategies among Ecuadorian university students, moving beyond broad binary sex comparisons to examine the underlying psychological mechanisms. Specifically, this study aimed to identify which distinct dimensions of gender beliefs drive the use of cognitive reappraisal and expressive suppression, to determine if biological sex moderates these relationships, and to evaluate whether these associations persist when accounting for relevant clinical and demographic covariates. The findings revealed that biological sex did not significantly differentiate the use of the investigated emotion regulation strategies, nor did it moderate the relationship between gender beliefs and these strategies. Instead, the internalisation of specific gender norms emerged as a robust predictor for both men and women. Non-egalitarian roles significantly and negatively predicted the use of both cognitive reappraisal and expressive suppression, whereas traditional roles positively predicted only expressive suppression. Furthermore, these associations remained highly significant even after controlling for covariates such as income, mental health history, and marital status.

The divergent predictive paths of non-egalitarian roles (GRAS 1) and traditional roles (GRAS 4) suggest that these subscales capture distinct dimensions of gender ideology that place differing psychological demands on the individual. A plausible explanation for these opposing contributions involves the distinction between abstract socio-political attitudes and active interpersonal behavioural scripts. The items comprising GRAS 1 assess structural, macro-level viewpoints regarding socioeconomic fairness, such as equal pay, equitable asset distribution, and joint marital decision-making. Endorsing non-egalitarian views on these structural items may reflect a rigid socio-political worldview that does not directly prescribe immediate personal behaviour, correlating instead with a generalised reduction in engagement in active emotion regulation. Conversely, the items in GRAS 4 capture highly specific, proximal behavioural mandates, including explicit domestic hierarchies, obedience to patriarchal authority, and strict gender-typed expectations. Adhering to these interpersonal rules likely functions as a demanding behavioural blueprint. To conform to these explicit scripts and avoid social sanctions, individuals may be actively compelled to regulate their affect, relying exclusively on expressive suppression to conceal counter-normative emotions. Thus, while non-egalitarian attitudes may represent a passive ideological stance that reduces active emotional management across strategies, traditional roles may serve as an active behavioural mandate that specifically necessitates reliance on expressive suppression.

These results contribute to an evolving conceptualisation of emotional processing, suggesting that the relationship between gender beliefs—specifically egalitarian versus traditional constructs—and emotion regulation may operate largely independently of an individual’s biological sex. Historically, much of the psychological literature has conflated biological sex with gendered socialisation, often presuming that men and women naturally diverge in their regulatory habits due to innate physiological differences or rigid, sex-specific social scripts (Chaplin, 2015; Hyde, 2014; Whittle et al., 2011). However, our findings suggest a more nuanced reality wherein the endorsement of egalitarian beliefs functions as a cognitive schema accessible to any individual. If a person, regardless of whether they are male or female, internalises egalitarian gender roles, they are equally likely to experience the associated psychological flexibility that facilitates adaptive emotional regulation. Conversely, rigid adherence to traditional roles appears to tether both men and women to internally mediated strategies like expressive suppression, whilst non-egalitarian roles constrict both emotional expression and cognitive reframing. This indicates that cognitive reappraisal and expressive suppression are not inherently male or female behaviours, but rather psychological tools whose deployment may correspond with the perceived social permissibility associated with one’s internalised gender role attitudes.

Consequently, the present data reinforce the premise that sociocultural belief systems serve as significant predictors of emotional regulation strategies, accounting for variance beyond biological sex (Brody, 1999; Else-Quest et al., 2012). When individuals evaluate an emotionally evocative event, their subsequent regulatory efforts are filtered through their learned cultural frameworks regarding appropriate emotional conduct (Ford and Mauss, 2015; Matsumoto et al., 2008). Individuals tightly bound by traditional roles often experience heightened discrepancy stress if their genuine affective states contradict societal expectations, thereby compelling them to suppress outward emotional expressions to avoid social penalisation (Berke et al., 2018; Levant and Richmond, 2007). The overarching influence of these internalised gender roles overshadows binary sex differences, aligning with recent evidence highlighting that social constructs and cognitive flexibility are primary drivers of emotional processing (García-Fernández et al., 2025; Rodas et al., 2022). The transition from traditional to egalitarian paradigms within Latin American contexts, particularly among young university populations, provides a unique environment where these cognitive schemas are actively being renegotiated (Nuñez et al., 2016).

Despite the clear evidence that biological sex did not differentiate the use of the investigated emotion regulation strategies within our sample, a pronounced epidemiological paradox remains. Women consistently exhibit a significantly higher global prevalence of internalising conditions, such as mood and anxiety disorders, compared to men (Steel et al., 2014). If men and women with similar gender role attitudes use cognitive reappraisal and expressive suppression at comparable rates, the disparity in psychopathological vulnerability requires further exploration. One plausible explanation is that our study, by focusing exclusively on cognitive reappraisal and expressive suppression, might not have evaluated the cognitive patterns of emotion regulation in sufficient detail. Previous research within the Ecuadorian context has demonstrated that whilst men and women might share similarities in certain regulatory domains, women often display a markedly higher propensity for maladaptive cognitive strategies, such as rumination and self-blame, particularly under conditions of high psychological distress (Alvarado-Zurita and Rodas, 2024). Rumination, characterised by a repetitive and passive focus on the symptoms of distress, is a well-established transdiagnostic risk factor for both depression and anxiety. Therefore, the equal utilisation of reappraisal or suppression does not necessarily equate to an overall identical emotional regulation profile, as other unmeasured, sex-differentiated cognitive strategies might be driving the epidemiological disparities in mental health outcomes.

To contextualise these findings, gender roles and their associated emotional regulation strategies can be viewed as adaptive responses to complex social environments (Geary, 2021). The contemporary shift towards egalitarian beliefs reflects an ongoing adaptation to modern societies, where rigid, sex-specific divisions of labour are no longer prerequisites for survival, thereby facilitating greater psychological and emotional flexibility (Eagly and Wood, 1999).

### 4.1 Limitations

The findings of this research must be interpreted within the context of several methodological limitations. Primarily, generalisability is constrained by the use of a convenience and snowball sample consisting exclusively of Ecuadorian university students. This demographic generally possesses higher educational attainment and may hold more progressive or egalitarian views compared to the broader Ecuadorian general population, potentially restricting the variance in gender role attitudes. Furthermore, the clear imbalance between women and men within the sample limits representativeness and weakens claims regarding sex differences. The operationalisation of gender is also narrow; the study explicitly measures gender role attitudes and does not capture gender identity, gender expression, or broader gendered experiences. The absence of an *a priori* power analysis represents an additional limitation. Interaction analyses require substantially larger samples to achieve adequate statistical power; therefore, the non-significant moderating effects of biological sex must be interpreted with caution, as they may reflect insufficient power rather than a true absence of an interaction. Secondly, the cross-sectional nature of the data precludes the establishment of causal relationships between gender beliefs and emotion regulation strategies; longitudinal designs are required to ascertain the temporal precedence of these variables. Additionally, the assessment of emotion regulation was limited to cognitive reappraisal and expressive suppression. The inclusion of a broader array of adaptive and maladaptive strategies, such as rumination, catastrophising, or acceptance, would provide a more comprehensive understanding of the emotional repertoire influenced by gender beliefs. Regarding covariates, the mental health history variable used was a crude, binary self-report that did not capture current symptoms, clinical severity, treatment status, or active psychological distress. Furthermore, the analytical approach involved multiple statistical tests, which inherently increases the risk of Type I error. Finally, the exclusive reliance on self-report measures raises concerns regarding shared-method variance and social desirability bias, particularly when assessing sensitive ideological constructs such as gender roles in a transitional cultural context.

In conclusion, the present study advances the literature on emotional processing by demonstrating that the internalisation of sociocultural gender beliefs better accounts for individual differences in emotion regulation. By establishing that non-egalitarian and traditional role endorsements independently drive the distinct deployment of cognitive reappraisal and expressive suppression, this research highlights the profound psychological impact of societal norms. Whilst these findings offer critical insights into the cognitive schemas governing emotional regulation, the enduring disparities in global mental health prevalence indicate that future research must delve deeper into more complex cognitive strategies and the pervasive influence of systemic social hierarchies. Ultimately, fostering egalitarian gender beliefs may serve as a vital, transdiagnostic target in clinical psychology, promoting greater psychological flexibility and more adaptive emotional functioning across diverse populations.

## Data Availability

The raw data supporting the conclusions of this article will be made available by the authors, without undue reservation.

## References

[ref1] Aldao A. Nolen-Hoeksema S. Schweizer S. (2010). Emotion-regulation strategies across psychopathology: a meta-analytic review. Clin. Psychol. Rev. 30, 217–237. doi: 10.1016/j.cpr.2009.11.004, 20015584

[ref2] Alvarado-Zurita P. Rodas J. A. (2024). Estrategias de regulación emocional cognitiva: Diferencias de género en Ecuador. Liberabit 30:e727. doi: 10.24265/liberabit.2024.v30n1.727

[ref3] American Psychological Association. (2017). Ethical Principles of Psychologists and Code of Conduct. Washington, DC: American Psychological Association. Available online at: http://www.apa.org/ethics/code/index.aspx (accessed June 3, 2026).

[ref4] Berke D. S. Reidy D. E. Gentile B. Zeichner A. (2019). Masculine discrepancy stress, emotion-regulation difficulties, and intimate partner violence. J. Interpers. Violence 34, 1163–1182. doi: 10.1177/0886260516650967, 27226013 PMC5861012

[ref5] Berke D. S. Reidy D. Zeichner A. (2018). Masculinity, emotion regulation, and psychopathology: a critical review and integrated model. Clin. Psychol. Rev. 66, 106–116. doi: 10.1016/j.cpr.2018.01.004, 29398184

[ref6] Brody L. R. (1999). Gender, Emotion, and the Family. Cambridge: Harvard University Press.

[ref7] Chaplin T. M. (2015). Gender and emotion expression: a developmental contextual perspective. Emot. Rev. 7, 14–21. doi: 10.1177/1754073914544408, 26089983 PMC4469291

[ref8] Eagly A. H. Wood W. (1999). The origins of sex differences in human behavior: evolved dispositions versus social roles. Am. Psychol. 54, 408–423. doi: 10.1037/0003-066X.54.6.408

[ref9] Else-Quest N. M. Higgins A. Allison C. Morton L. C. (2012). Gender differences in self-conscious emotional experience: a meta-analysis. Psychol. Bull. 138, 947–981. doi: 10.1037/a0027930, 22468881

[ref10] Ford B. Q. Mauss I. B. (2015). Culture and emotion regulation. Curr. Opin. Psychol. 3, 1–5. doi: 10.1016/j.copsyc.2014.12.004, 25729757 PMC4341898

[ref11] Fox J. Weisberg S. (2019). An R Companion to Applied Regression. 3rd Edn Thousand Oaks: Sage.

[ref12] García-Fernández M. Fuentes-Sánchez N. Escrig M. A. Eerola T. Pastor M. C. (2025). Gender, emotion regulation, and cognitive flexibility as predictors of depression, anxiety, and affect in healthy adults. Curr. Psychol. 44, 5685–5694. doi: 10.1007/s12144-024-07240-6

[ref13] Gardener E. K. T. Carr A. R. MacGregor A. Felmingham K. L. (2013). Sex differences and emotion regulation: an event-related potential study. PLoS One 8:e73475. doi: 10.1371/journal.pone.0073475, 24204562 PMC3813629

[ref14] Geary D. C. (2021). Male, Female: The Evolution of Human Sex Differences. 3rd Edn Washington, DC: American Psychological Association.

[ref15] Goubet K. E. Chrysikou E. G. (2019). Emotion regulation flexibility: gender differences in context sensitivity and repertoire. Front. Psychol. 10:935. doi: 10.3389/fpsyg.2019.00935, 31143142 PMC6521736

[ref16] Gross J. J. (2015a). Emotion regulation: current status and future prospects. Psychol. Inq. 26, 1–26. doi: 10.1080/1047840X.2014.940781

[ref17] Gross J. J. (2015b). The extended process model of emotion regulation: elaborations, applications, and future directions. Psychol. Inq. 26, 130–137. doi: 10.1080/1047840X.2015.989751

[ref18] Gross J. J. John O. P. (2003). Individual differences in two emotion regulation processes: implications for affect, relationships, and well-being. J. Pers. Soc. Psychol. 85, 348–362. doi: 10.1037/0022-3514.85.2.348, 12916575

[ref19] Hartung C. M. Lefler E. K. (2019). Sex and gender in psychopathology: DSM–5 and beyond. Psychol. Bull. 145, 390–409. doi: 10.1037/bul0000183, 30640497

[ref20] Hyde J. S. (2014). Gender similarities and differences. Annu. Rev. Psychol. 65, 373–398. doi: 10.1146/annurev-psych-010213-115057, 23808917

[ref21] Instituto Nacional de Estadística y Censos. (2025). Boletín técnico N° 01-2025-IPC: Índice de precios al consumidor. Available online at: https://www.ecuadorencifras.gob.ec (accessed June 3, 2026).

[ref22] Levant R. F. Richmond K. (2007). A review of research on masculinity ideologies using the male role norms inventory. J. Men’s Stud. 15, 130–146. doi: 10.3149/jms.1502.130, 41656320

[ref23] Matsumoto D. Yoo S. H. Fontaine J. (2008). Mapping Expressive Differences Around the World: The Relationship Between Emotional Display Rules and Individualism Versus Collectivism. Journal of Cross-Cultural Psychology. 39, 55–74. doi: 10.1177/0022022107311854

[ref24] McRae K. Ochsner K. N. Mauss I. B. Gabrieli J. J. D. Gross J. J. (2008). Gender differences in emotion regulation: an fMRI study of cognitive reappraisal. Group Process. Intergroup Relat. 11, 143–162. doi: 10.1177/1368430207088035, 29743808 PMC5937254

[ref25] Moreta-Herrera R. Dominguez-Lara S. Rodas J. A. Sánchez-Guevara S. Montes-De-Oca C. Rojeab-Bravo B. . (2022). Examining psychometric properties and measurement invariance of the emotion regulation questionnaire in an Ecuadorian sample. Psychol. Thought 15, 57–74. doi: 10.37708/psyct.v15i2.634

[ref26] Nolen-Hoeksema S. (2012). Emotion regulation and psychopathology: the role of gender. Annu. Rev. Clin. Psychol. 8, 161–187. doi: 10.1146/annurev-clinpsy-032511-143109, 22035243

[ref27] Nolen-Hoeksema S. Aldao A. (2011). Gender and age differences in emotion regulation strategies and their relationship to depressive symptoms. Personal. Individ. Differ. 51, 704–708. doi: 10.1016/j.paid.2011.06.012

[ref28] Nuñez A. González P. Talavera G. A. Sanchez-Johnsen L. Roesch S. C. Davis S. M. . (2016). Machismo, marianismo, and negative cognitive-emotional factors: findings from the Hispanic community health study/study of Latinos sociocultural ancillary study. J. Lat. Psychol. 4, 202–217. doi: 10.1037/lat0000050, 27840779 PMC5102330

[ref29] Pejičić M. (2024). Unveiling gendered scripts about basic emotions and their implications. Facta Univ. Ser. Philos. Sociol. Psychol. Hist. 23, 105–119. doi: 10.22190/FUPSPH240823009P

[ref30] R Core Team. (2024). R: A Language and Environment for Statistical Computing R Foundation for Statistical Computing Vienna. Available online at: https://www.R-project.org/ (accessed June 3, 2026).

[ref31] Revelle W. (2026). psych: Procedures for Psychological, Psychometric, and Personality Research Northwestern University Evanston (R package version 2.6.3). Available online at: https://CRAN.R-project.org/package=psych (accessed June 3, 2026).

[ref32] Rodas J. A. Jara-Rizzo M. F. Greene C. M. Moreta-Herrera R. Oleas D. (2022). Cognitive emotion regulation strategies and psychological distress during lockdown due to COVID -19. Int. J. Psychol. 57, 315–324. doi: 10.1002/ijop.12818, 34747019 PMC8652999

[ref33] Steel Z. Marnane C. Iranpour C. Chey T. Jackson J. W. Patel V. . (2014). The global prevalence of common mental disorders: a systematic review and meta-analysis 1980-2013. Int. J. Epidemiol. 43, 476–493. doi: 10.1093/ije/dyu038, 24648481 PMC3997379

[ref34] Whittle S. Yücel M. Yap M. B. Allen N. B. (2011). Sex differences in the neural correlates of emotion: evidence from neuroimaging. Biol. Psychol. 87, 319–333. doi: 10.1016/j.biopsycho.2011.05.003, 21600956

[ref35] Wickham H. François R. Henry L. Müller K. Vaughan D. (2026). dplyr: a grammar of data manipulation. R Package Version 1.2.0. Available online at: https://CRAN.R-project.org/package=dplyr (accessed June 3, 2026).

[ref36] Zeyneloğlu S. Terzioğlu F. (2011). Development and psychometric properties of the gender roles attitude scale. Hacettepe Univ. Egit. Fak. Derg., 40, 409–420.

